# Hepatitis C Seroprevalence and Associated Risk Factors, Anyang, China

**DOI:** 10.3201/eid1511.0900263

**Published:** 2009-11

**Authors:** Fangfang Liu, Ke Chen, Zhonghu He, Tao Ning, Yaqi Pan, Hong Cai, Yang Ke

**Affiliations:** Ministry of Education Key Laboratory, Beijing, People’s Republic of China (F. Liu, K. Chen, T. Ning, Y. Pan, H. Cai, Y. Ke); Peking University School of Oncology, Beijing (F. Liu, K. Chen, T. Ning, Y. Pan, H. Cai, Y. Ke); Beijing Cancer Hospital and Institute, Beijing (F. Liu, K. Chen, T. Ning, Y. Pan, H. Cai, Y. Ke); Peking University School of Public Health, Beijing (Z. He); 1 These authors contributed equally to this article.

**Keywords:** HCV, hepatitis, infection, risk factor, esophageal balloon examination, viruses, dispatch

## Abstract

Hepatitis C virus screening was conducted among 8,226 residents 25–65 years of age in 4 counties of China; virus prevalence was 0.9%. A subsequent case–control study indicated blood transfusion (odds ratio [OR] 4.55), esophageal balloon examination (OR 3.78), and intravenous injection (OR 5.83) were associated with infection.

Hepatitis C virus (HCV) infection shows clear differences in prevalence among geographic regions, according to World Health Organization data (*1*). HCV prevalence also varies over time and with behavioral changes (*2*,*3*). HCV prevalence in the People’s Republic of China nationwide was estimated at 3.2% in a 1992 survey (*4*), but studies have reported regional prevalence rates ranging from 0% to 31.9% (*5*–*7*). In developing countries, transmission of HCV typically results primarily from iatrogenic factors, such as blood transfusion and inadequate sterilization or reuse of medical equipment (*8*), but in industrialized countries, risk resulting from these factors has been greatly reduced (*9*,*10*).

In an esophageal endoscopic survey (2006–2008) in Anyang, Henan Province, China, blood screening for the HCV antibody was carried out in all participants. Because HCV infection is an important public health issue, a case–control study was performed among HCV-positive case-patients with matched controls to evaluate risk factors for HCV infection in the area where the esophageal endoscopic survey was conducted.

## The Study

An endoscopic survey (2006–2008) for esophageal cancer was conducted in 8 villages of 4 counties of Anyang, Henan Province, China (Figure). The villages were selected on the basis of population size, location, and village administrative capabilities. A total of 10,240 permanent residents were eligible for the survey; 8,226 (80.3%) persons 25–65 years of age (median age 42.0, M:F sex ratio 1.00:1.18) without cardiovascular or psychological diseases were interviewed and had blood drawn. A total of 74 participants were seropositive for HCV in this screening; 69 of them were enrolled in the subsequent case–control study. Each seropositive person was matched with 3 negative controls randomly selected from seronegative participants (2.5%) of the same village, gender, and age (±2 years). A questionnaire regarding lifetime risk factors for HCV infection was given to HCV seropositive case-patients and matched controls. The Institutional Review Board of the School of Oncology, Peking University, China, approved this study. Informed consent was obtained from all participants.

**Figure:**
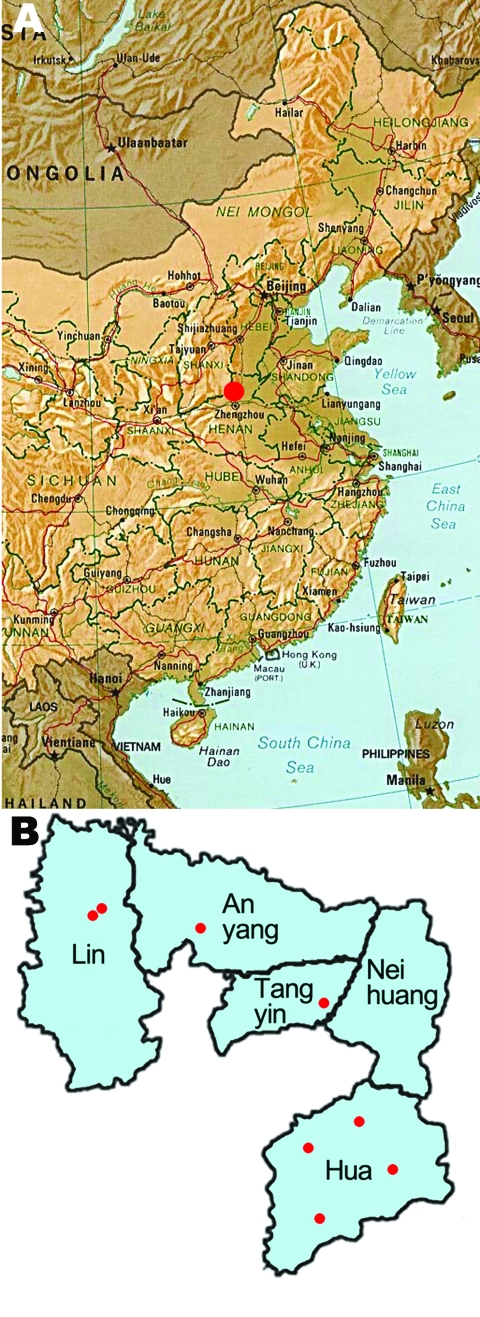
A) Map of eastern China showing the location of Anyang (red dot). B) Villages (red dots) targeted in the 2006–2008 hepatitis C virus prevalence study.

Serum samples were separated from blood samples by centrifugation and tested for HCV in the Anyang Cancer Hospital for case-patients from Anyang, Lin, and Tangyin counties, and in the Hua County Hospital for case-patients from Hua County. ELISAs were performed to evaluate for HCV antibody (Diagnostic Kit for HCV ELISA 3.0; AutoBio Co., Zhengzhou, China). HCV-positive case-patients submitted an additional blood sample for a confirmatory HCV test in Anyang Cancer Hospital. Positivity for HCV antibody in these 2 tests was independently confirmed at Beijing Friendship Hospital by ELISA (Diagnostic Kit HCV 3.0; Abbott GmbH & Co. KG, Wiesbaden-Delkenheim, Germany). We determined the positive predictive value of the anti-HCV tests conducted in China to be 98.3% by using the testing result of the Abbott diagnostic kit as a standard. Assays were monitored with internal and external quality controls.

We examined group differences using the χ^2^ test. Univariate and multivariate conditional logistic regression were used to identify significant factors for HCV infection. Data entry and statistical analysis were conducted using Epi Data 3.1 (www.cdc.gov/epiinfo) and SAS 9.1.3 (SAS, Cary, NC, USA); p<0.05 was considered significant.

Seventy-four (0.9%) of 8,266 participants were HCV positive. Prevalence of infection varied significantly with age (p<0.001) and county of origin (p<0.001) but not with gender (Table 1). HCV prevalence was significantly higher in participants >50 years of age. Prevalence was highest in Lin County (2.8%), followed by Anyang County (0.7%), Tangyin County (0.4%), and Hua County (0.2%) (Table 1). The Lin County >50-year-old group showed an 18.25-fold higher risk for HCV infection (95% confidence interval [CI] 9.29–35.83) when compared with non–Lin County participants <50 years of age (data not shown).

**Table 1:** Demographic distribution and HCV infection status of participants (n = 8,226) in an esophageal endoscopic survey for HCV, Anyang, China, 2006–2008*

Variable	Total no. (%)	HCV-positive, no. (%)	p value
Age, y			
<50	5,766 (70.1)	37 (0.6)	<0.001
>50	2,460 (29.9)	37 (1.5)	
Sex			
M	3,782 (46.0)	31 (0.8)	0.479
F	4,444 (54.0)	43 (1.0)	
County			
Hua	4,022 (48.9)	7 (0.2)	<0.001
Anyang	838 (10.2)	6 (0.7)	
Lin	1,980 (24.1)	55 (2.8)	
Tangyin	1,386 (16.8)	6 (0.4)	

*HCV, hepatitis C virus.

Univariate conditional logistic regression was used to evaluate possible risk factors based on information collected from the 69 HCV-positive participants and 207 matched controls. Transfusion with blood and blood products, intravenous injection, and procedures including Caesarean section, acupuncture, gastroscopy, and esophageal balloon examination were associated with higher risk for HCV infection. No instances of hemodialysis, organ transplantation, drug use, or homosexual behavior were identified. However, when these risk factors were analyzed in a multivariate model, only blood transfusion (odds ratio [OR] 4.55, 95% CI 1.34–15.42), intravenous injection (OR 5.83, 95% CI 2.66–12.80), and esophageal balloon examination (OR 3.78, 95% CI 1.32–10.79) were significant (Table 2). A repeat analysis of participants from Lin County produced almost identical results (data not shown).

**Table 2:** Conditional logistic analysis of risk factors associated with hepatitis C virus infection, Anyang, China, 2006–2008*

Risk factor†	Response	Total no.	HCV negative, no. (%)	HCV positive, no. (%)	OR (95% CI)	Adjusted OR (95% CI)
Alcohol consumption	No	258	195 (94.2)	63 (91.3)	1	
	Yes	18	12 (5.8)	6 (8.7)	1.71 (0.54–5.39)	
Blood donation	No	261	199 (96.1)	62 (89.9)	1	
	Yes	15	8 (3.9)	7 (10.1)	3 (0.99–9.09)	
Blood transfusion	No	248	197 (95.2)	51 (73.9)	1	1
	Yes	28	10 (4.8)	18 (26.1)	6.32‡ (2.73–14.6)	4.55§ (1.34–15.42)
Blood products transfusion	No	249	191 (92.3)	58 (84.1)	1	1
	Yes	27	16 (7.7)	11 (15.9)	2.49§ (1.03–6.05)	0.99 (0.28–3.5)
Intramuscular injection	No	39	32 (15.5)	7 (10.1)	1	
	Yes	237	175 (84.5)	62 (89.9)	1.77 (0.69–4.59)	
Intravenous injection	No	153	136 (65.7)	17 (24.6)	1	1
	Yes	123	71 (34.3)	52 (75.4)	6.75‡ (3.41–13.36)	5.83‡ (2.66–12.8)
Visited a dentist	No	136	103 (49.8)	33 (47.8)	1	
	Yes	140	104 (50.2)	36 (52.2)	1.09 (0.62–1.9)	
Had surgery	No	210	171 (82.6)	39 (56.5)	1	1
	Yes	66	36 (17.4)	30 (43.5)	3.78‡ (2.01–7.1)	2.29 (0.92–5.71)
Shared nail clippers	No	66	51 (24.6)	15 (21.7)	1	
	Yes	210	156 (75.4)	54 (78.3)	1.17 (0.62–2.23)	
No. sexual partners	1	262	194 (93.7)	68 (98.6)	1	
	>1	14	13 (6.3)	1 (1.4)	0.21 (0.03–1.66)	
Pierced ears	No	173	129 (62.3)	44 (63.8)	1	
	Yes	103	78 (37.7)	25 (36.2)	0.88 (0.4–1.96)	
Acupuncture	No	215	168 (81.2)	47 (68.1)	1	
	Yes	61	39 (18.8)	22 (31.9)	2.15§ (1.11–4.13)	1.61 (0.69–3.76)
Gastroscopy	No	246	190 (91.8)	56 (81.2)	1	
	Yes	30	17 (8.2)	13 (18.8)	2.92§ (1.27–6.72)	2.06 (0.63–6.7)
Esophageal balloon examination	No	240	190 (91.8)	50 (72.5)	1	
	Yes	36	17 (8.2)	19 (27.5)	5.95‡ (2.44–14.5)	3.78§ (1.32–10.79)

*HCV, hepatitis C virus; OR, odds ratio; CI, confidence interval. †Blood transfusion, blood products transfusion, intravenous injection, operation, acupuncture, gastroscopy, and esophageal balloon examination were included in the multivariate model. ‡p<0.01. §p<0.05.

## Conclusions

In this 2006–2008 study, overall HCV prevalence was 0.9%, with prevalence highest in the >50-year-old group of Lin County (4.7%). In a 2000 study of 55- to 84-year-old Lin County residents, the prevalence of HCV was 9.6% (*7*). Several possible reasons could explain these differences. One is that the average age in the previous study (range 64–84 years) was greater than that in our study (range 25–65 years); older persons were more likely to be infected in both the previous study and our study. The time interval between these 2 studies might also have contributed to the change in HCV prevalence.

A case–control study was performed to identify HCV infection risk factors. Blood transfusion and medical intravenous injection with reusable glass syringes and needles, which are established HCV risk factors, were associated with HCV infection (*10*,*11*). In addition, esophageal balloon examination, a less commonly identified route of HCV infection, also increased the risk for HCV infection. In the recent past (1980–2000), esophageal balloon examination, which was designed for early cytologic detection of esophageal lesions, was relatively common in China for diagnosis and screening of persons in high-risk populations (*12*). In this technique, the patient swallows a balloon covered with a cotton net. The balloon is inflated within the patient’s stomach. Exfoliated esophageal cells are then scraped off the mucosa by pulling out the balloon. Bleeding of esophageal mucosa can occur. The balloon and cotton net were designed to be nonreusable. Nonetheless, on some occasions, balloons were reused after manual cleaning. This technique is no longer widely used; however, Lin County is a high-risk area for esophageal cancer. Screening for esophageal cancer using balloon examination was performed in this region before 2000. Reuse of balloons and occasional bleeding during the procedure may have caused transmission of HCV in this population.

A nationwide survey for HCV infection in China was performed in 1992; prevalence was 3.1% for residents in rural areas. However, prevalence of viral infection was not consistent across regional populations, similar to what was observed in the present study (*4*). On the basis of these regional differences in HCV distribution and the potential risk factors identified in this study, we strongly suggest that unregulated medical procedures may confer substantial risk for HCV spread.

Chronic infection will develop in ≈75%–85% of persons infected with HCV, and cirrhosis of the liver will develop in up to 20% of chronically infected persons. Hepatocellular carcinoma will develop in ≈3%–4% of patients with HCV-associated cirrhosis each year (*13*–*15*). Given the serious social and economic effect of this HCV epidemic, strengthening administrative regulation of medical practice, especially in rural areas, and providing appropriate education to the public about HCV infection and its transmission should be given higher priority in public health policy.
